# OnabotulinumtoxinA Dosing, Disease Severity, and Treatment Benefit in Patients With Cervical Dystonia: A Cohort Analysis From CD PROBE

**DOI:** 10.3389/fneur.2022.914486

**Published:** 2022-06-30

**Authors:** Pinky Agarwal, Richard Barbano, Henry Moore, Marc Schwartz, Aleksej Zuzek, Marjan Sadeghi, Atul Patel

**Affiliations:** ^1^Evergreen Medical Center, Kirkland, WA, United States; ^2^University of Washington, Seattle, WA, United States; ^3^University of Rochester, Rochester, NY, United States; ^4^University of Miami—Miller School of Medicine, Miami, FL, United States; ^5^MS Biostatistics LLC, Clermont, FL, United States; ^6^AbbVie, Irvine, CA, United States; ^7^Kansas City Bone & Joint Clinic, Overland Park, KS, United States; ^8^University of Missouri, Kansas City, MO, United States

**Keywords:** cervical dystonia, onabotulinumtoxinA, treatment benefit, disease severity, dosing

## Abstract

**Introduction:**

The Cervical Dystonia Patient Registry for Observation of OnabotulinumtoxinA Efficacy (CD PROBE) study (ClinicalTrials.gov identifier: NCT00836017), a multicenter, prospective, observational registry, was designed to identify real-world practices and outcomes for patients with cervical dystonia (CD) treated with onabotulinumtoxinA (onabotA). This secondary analysis from CD PROBE aims to determine the impact of presentation subtype on onabotA utilization and CD severity.

**Materials and Methods:**

The study cohort includes those who completed all 3 treatments, 4 office visits, and had data recorded for all assessments. Patient outcomes were assessed with the Cervical Dystonia Impact Profile (CDIP-58), Toronto Western Spasmodic Torticollis Rating Scale (TWSTRS), and determination of CD severity. Treatment interval, dose, and adverse events (AEs) were also recorded. Data were stratified according to prior exposure to botulinum toxins (BoNTs) and analyzed with descriptive statistics.

**Results:**

Torticollis was the most common presentation subtype in the study cohort (*N* = 350); the proportion of patients with torticollis was highest in those with severe disease. At each treatment, between 40.7 and 65.2% of those categorized as severe shifted to moderate or mild severity after treatment. Sustained improvements in CDIP-58 and TWSTRS were observed regardless of prior exposure to BoNTs. Dosing of onabotA generally increased from injection 1 to injection 3 and tended to be lower for patients naïve to BoNT. Median time interval between injections for the study cohort was 94.0 to 97.5 days. The most common AEs (dysphagia, muscular weakness) and injection intervals were similar between naïve vs. non-naïve patients; there were no serious treatment-related AEs.

**Conclusions:**

This secondary cohort analysis from CD PROBE demonstrates that three repeat treatments with onabotA at intervals consistent with labeling attenuated disease severity and neck pain, resulting in sustained improvements in physician- and patient-reported outcomes. No new safety signals were identified.

## Introduction

Cervical dystonia (CD) is a movement disorder characterized by the involuntary contraction of cervical muscles in the neck and upper shoulders, resulting in abnormal head movements and postures (1, 2). Isolated idiopathic CD (3) is the most common focal dystonia (1, 3–5). Although CD can occur at any age (1), the prevalence of CD rises from <30 cases per million in the general population (6, 7) to more than 700 cases per 100,000 individuals in the population of those over 50 years of age (8). Service-based studies have shown a point prevalence for CD ranging from 5.0 to 17.8 cases per 100,000 individuals (7, 9). CD has a mean age of onset in the early 40s (1), affecting women nearly twice as often as men (3, 10).

Adult-onset CD disrupts workplace productivity during years that are typically among the most productive (11), resulting in higher absenteeism and presenteeism (functioning at less than full capacity while present in the workplace) (12). Pain is the most frequently reported symptom of patients with CD, affecting between 70 and 90% of patients (1, 11, 13–16). Overall, the functional impairment and pain experienced by patients with CD have been shown to negatively impact their quality of life (11, 17–19).

Currently, focal injections of botulinum toxins (BoNTs) are recommended as first-line treatment for CD, based on evidence supporting the efficacy of BoNT/A (and BoNT/B) in the treatment of CD (20, 21). Other treatments for CD, which have been used with varying degrees of effectiveness, include pharmacologic therapies (anticholinergics, benzodiazepines, and baclofen), chemodenervation with injections of phenol, deep brain stimulation, or selective peripheral nerve denervation (22, 23).

Focal injections of BoNTs decrease excessive muscle contractions by inhibiting the release of acetylcholine into the synaptic cleft of the motor neuron, thus blocking neuromuscular transmission (24–26). Studies have shown that BoNT/A inhibits release of neuropeptides CGRP, substance P (27–30), and glutamate (31), and downregulates the expression of nociceptive ion channels TRPV1 and TRPA1 on nerve membranes (27, 28).

Although clinical trials have demonstrated the safety and efficacy of BoNTs for the treatment of CD, real-world evidence related to clinical factors such as disease severity, dosing, clinical presentation, and prior exposure to BoNTs can provide important information to help inform treatment decisions (32, 33). The Cervical Dystonia Patient Registry for Observation of onabotulinumtoxinA Efficacy (CD PROBE) was a multicenter, prospective, observational registry designed to identify patient- and clinician-reported outcomes from patients with CD after 3 treatment cycles with onabotulinumtoxinA (onabotA) (11, 32, 34). Primary results from CD PROBE demonstrate the effectiveness and safety of onabotA treatment in patients with CD (35). This secondary analysis of data from CD PROBE aims to evaluate the impact of presentation subtype and disease severity on onabotA utilization, stratified by prior exposure to BoNT. Since this study is focused on real-world data, there may have been a lack of control over study elements and confounding factors. Data from this secondary analysis, combined with controlled trial data, will help inform treatment of CD with onabotA. These real-world data describe patients who adhere to treatment by disease severity, subtype, and prior BoNT exposure.

## Materials and Methods

### Study Design and Participants

CD PROBE, an observational, multicenter, prospective clinical registry in the United States (US) (ClinicalTrials.gov identifier: NCT00836017), was designed to identify real-world outcomes in patients with CD after treatment with onabotA. Prior to enrolling any patients in the study, the institutional review board at each participating site approved the study protocol. Informed consent was obtained from each patient before performance of any study procedure.

The methods used in the CD PROBE study have been detailed in previous publications (32, 35) and are briefly summarized here. Participants were drawn from 82 centers and 88 physicians in the US between Jan. 12, 2009, and Aug. 31, 2012. Physicians with patients eligible for inclusion in this study were specialists in either neurology, pain, or physical medicine and rehabilitation. Patients eligible for inclusion had a diagnosis of CD and were viewed as candidates for BoNT therapy by their physician. Eligible patients were either new to a physician's practice, were new to BoNT treatment, or had not been treated with BoNT in a previous clinical trial for ≥16 weeks. Patients who completed 3 treatments and all study assessments (including assessments at the final office visit) were included in this secondary analysis.

### Treatments and Follow-Up Visits

**Figure 1:**
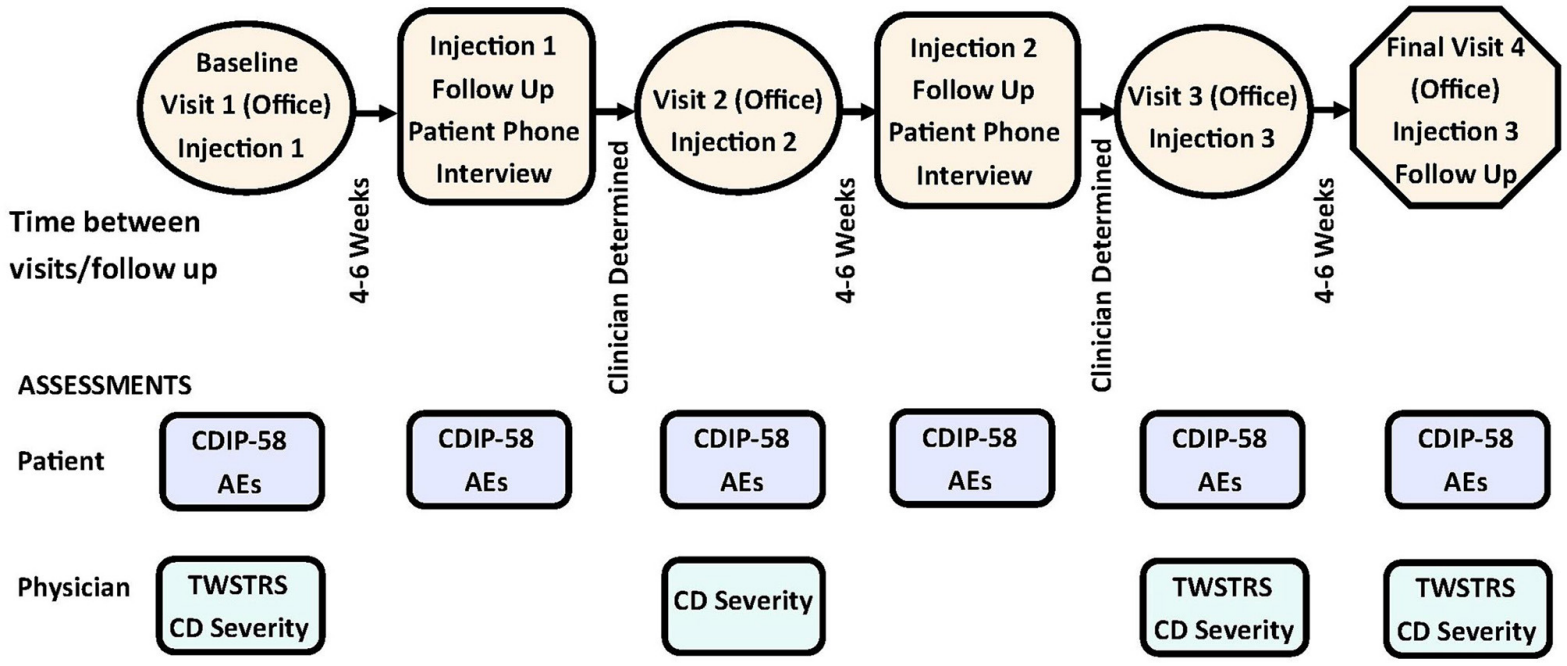
Study design of CD PROBE and timing of physician- and patient-reported assessments. AEs, adverse events; CD, cervical dystonia; CDIP-58, Cervical Dystonia Impact Profile-58; TWSTRS, Toronto Western Spasmodic Torticollis Scale. Figure is adapted with permission from Jankovic et al. (32).

Patients enrolled in this study received up to 3 treatments with onabotA (Figure 1). Patients received treatment at office visit 1 (baseline), visit 2, and visit 3. The muscles and dosage injected were at the discretion of the treating physician. Treatment intervals were determined by the physician, based on patients' clinical characteristics and clinical necessity. Patients were assessed during office visits during which they received treatment with onabotA, with the exception of the final office visit (visit 4). The final office visit involved assessment without concomitant treatment with onabotA. Patients were assessed between treatments via phone interviews 4–6 weeks after each treatment (Figure 1).

### Outcomes

This secondary analysis aimed to determine changes in CD severity after each treatment and identify dosages used in treating patients with various presentation subtypes and with differing degrees of CD severity, stratified by prior exposure to BoNT. We examined changes in parameters related to quality of life in those naïve and those non-naïve to BoNT treatment. Safety and tolerability were also assessed during patient visits and phone interviews.

### Assessments

Physicians assessed baseline characteristics and treatment outcomes with the CD-specific Toronto Western Spasmodic Torticollis Rating Scale (TWSTRS) (34) and by physician estimation of CD severity. TWSTRS, a validated assessment of CD, provides subscores for pain (range, 0–20), severity (range, 0–35), disability (range, 0–30), as well as a total score (range, 0–85) (36–38), with reduced scores indicative of improvement. Assessments of CD severity (mild, moderate, or severe) were based on the physician's determination of the most severe case of CD in their experience or estimation.

Patient-reported outcomes were assessed with the Cervical Dystonia Impact Scale (CDIP-58) (39) at baseline and after treatment. The CDIP-58, originally validated through mail-in surveys, was administered to patients in this study by phone. It contains 8 subscales, each ranging from 0 to 100. The subscales include evaluation of head and neck symptoms, pain and discomfort, upper limb activities, walking, sleep, annoyance, mood, and psychosocial functioning (39), with a lower score indicative of improvement in these measures which are designed to assess health and quality of life.

### Safety

Adverse events (AEs) were documented, including the date of AE onset and resolution, if applicable. AE severity (mild, moderate, or severe), frequency, duration, remedial actions, relationship to study treatment, and outcomes were evaluated and documented.

### Statistical Analysis

Descriptive statistics were used to analyze baseline and post-treatment outcomes in the cohort of patients who completed 3 treatments and 4 office visits, with data on record for all assessments. The cohort was further stratified according to prior BoNT treatment that may have occurred more than 16 weeks before enrollment in CD PROBE. Inferential statistics (paired *t*-tests) examined inter-treatment differences in dosing at the individual patient level, with Bonferroni adjustment for multiple comparisons. All analyses were performed with R version 4.1 or later (The R Foundation for Statistical Computing, http://www.rproject.org/).

## Results

### Patient Demographics

A total of 1046 patients were enrolled in the CD PROBE registry (35). The cohort included in this secondary analysis (*N* = 350) consists of patients who completed 3 treatments and 4 office visits, with data recorded for all study assessments (Table 1). Of this group, 212 were naïve to BoNT, and 138 were non-naïve to BoNT at enrollment (Supplementary Table 1). Of those that withdrew from the study after the third treatment (prior to the last office visit, *n* = 134), the majority (*n* = 110) were lost to follow up. The mean age of the study cohort at baseline was 57.3 years, with 74.9% female, 94.6% white, and 60.6% naïve to BoNT (Table 1). Demographics and CD history were similar between naïve and non-naïve patients (Supplementary Table 1). Baseline CD severity was recorded as mild, moderate, or severe in 32.6, 54.3, and 13.1% of patients, respectively (Table 1). The mean (standard deviation [SD]) time from disease onset to diagnosis was 5.1 years [7.7].

**Table 1:** Baseline demographics and disease characteristics.

**Total**	**(*N* = 350)**
**Age, years**	
*Mean (SD)*	57.3 (14.7)
**Female**, ***n*** **(%)**	262 (74.9)
**Race/ethnicity**, ***n*** **(%)**	
*White*	331 (94.6)
*Hispanic*	8 (2.3)
*Asian*	6 (1.7)
*Black*	5 (1.4)
**Prior BoNT exposure**, ***n*** **(%)**	138 (39.4)
**CD severity**, ***n*** **(%)**	
*Mild*	114 (32.6)
*Moderate*	190 (54.3)
*Severe*	46 (13.1)
**Predominant CD presentation subtype**, ***n*** **(%)**	
*Torticollis*	186 (53.1)
*Laterocollis*	122 (34.9)
*Anterocollis*	16 (4.6)
*Retrocollis*	14 (4.0)
*Other*	12 (3.4)

*BoNT, botulinum toxin; CD, cervical dystonia; SD, standard deviation*.

**Figure 2:**
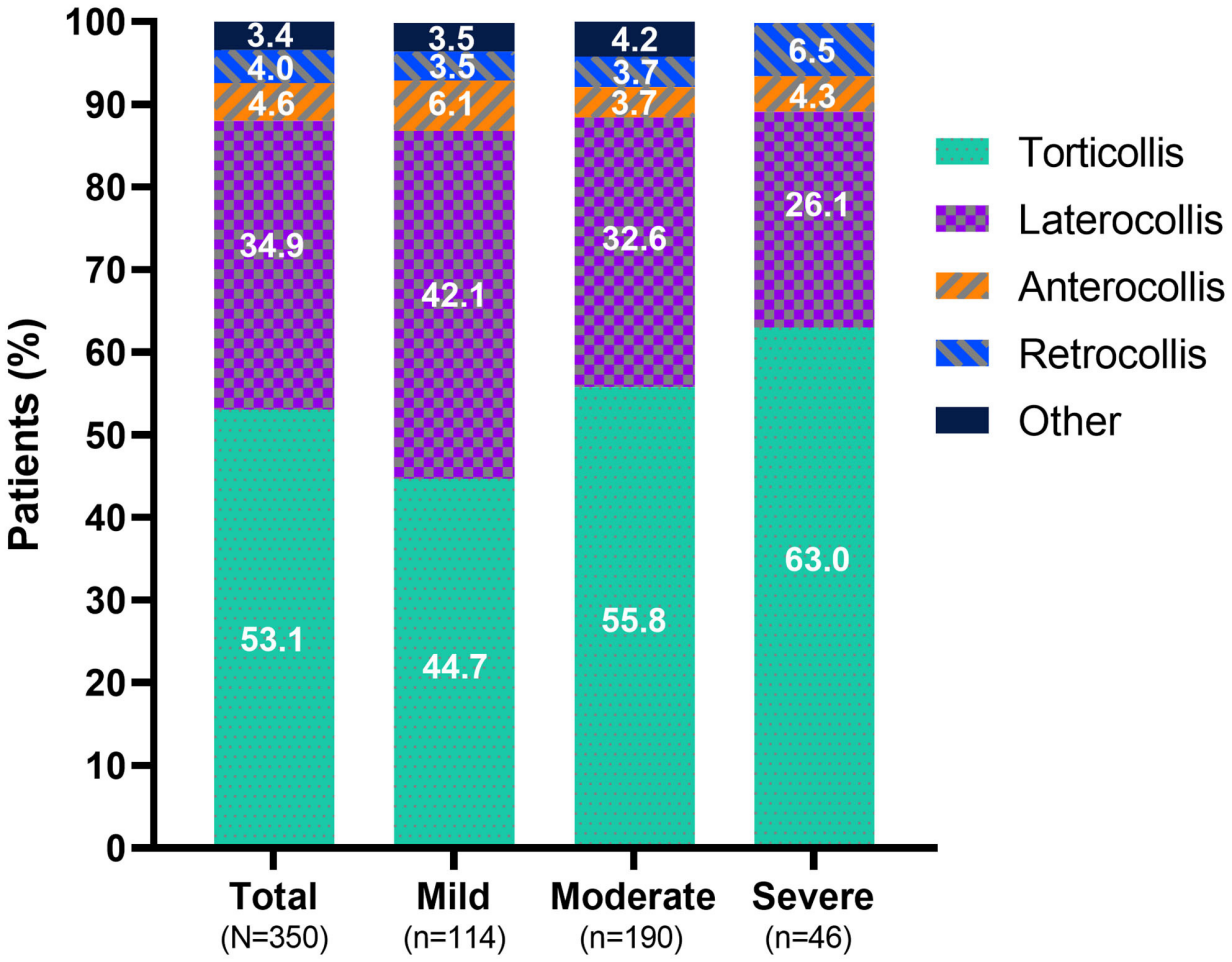
Predominant presentation subtype at baseline. The category “other” consists of 9 patients with no specific subtype mentioned and one case of each of the following subtypes: anterior lateral shift, lateral shift, and sagittal shift.

Regardless of disease severity or prior exposure to BoNTs, torticollis was the most common presentation subtype, followed by laterocollis (Figure 2). The proportion of individuals with torticollis was relatively higher in those with severe CD than those with milder CD. Of the 12 patients with CD subtypes listed as “other” in Figure 2, 9 cases had no subtype information; of the 3 that had subtype information on record, 1 indicated anterior lateral shift, 1 a lateral shift, and 1 a sagittal shift.

### Dosing

**Figure 3:**
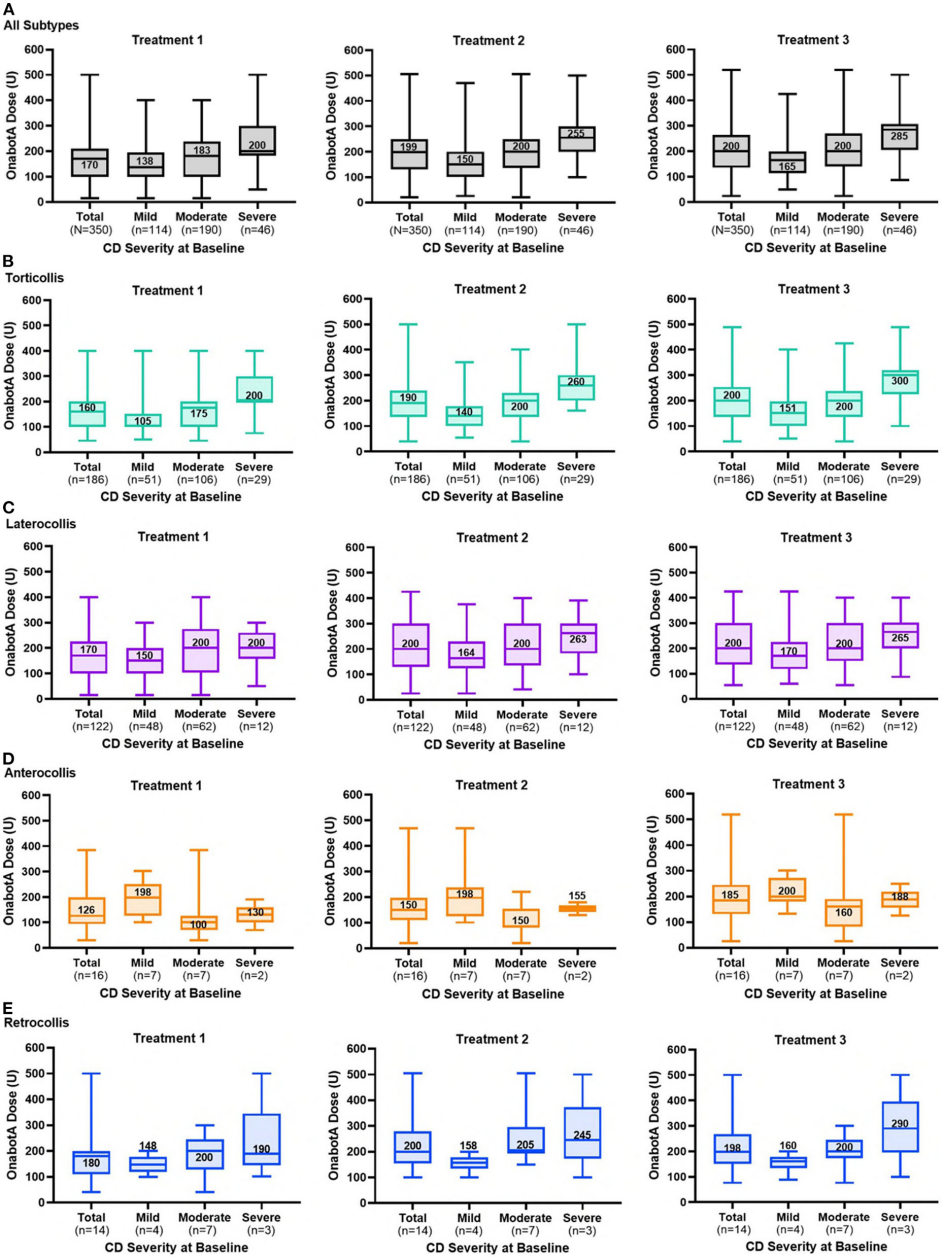
OnabotulinumtoxinA dose by CD severity at baseline for each treatment session for **(A)** all patients and those with **(B)** torticollis, **(C)** laterocollis, **(D)** anterocollis, and **(E)** retrocollis. The line within each box and associated number are median values; top and bottom of the box, interquartile range; whiskers, minimum and maximum.

For all types of CD combined, dosing of onabotA increased from injection 1 to injection 3 (Figure 3A), with a median dose for each injection ranging from 138 U to 165 U (mild), 183 U to 200 U (moderate), and 200 U to 285 U (severe). OnabotA doses tended to be lower in naïve than non-naïve patients. The median dose for all CD subtypes combined, stratified by prior exposure to BoNTs, ranged from 101 U to 150 U (naïve) vs. 200 U (non-naïve) for mild CD; 140 U to 193 U (naïve) vs. 225 U to 240 U (non-naïve) for moderate CD; and 195 U to 235 U (naïve) vs. 254 U to 300 U (non-naïve) for severe CD. The median onabotA dose used to treat torticollis or laterocollis in the study population increased over time for those who had mild or severe CD at baseline (Figures 3B,C). The median dose for anterocollis and retrocollis varied over time, with a relatively fewer number of patients in either of these two groups (Figures 3D,E) compared to the number of patients with torticollis or laterocollis. Retrocollis and anterocollis are typically less common presentation subtypes; in this study, patients with a combination of subtypes were classified according to their predominant or primary presentation subtype, resulting in relatively few patients with purely anterocollis or retrocollis. The mean pairwise difference in dose for each patient (all presentation subtypes combined) increased significantly from the first to the second injection for those presenting with mild, moderate, or severe CD (*P* = 0.0003, *P* = 0.0004, and *P* = 0.0014, respectively); the dose increased significantly from the first to the final (third) injection, regardless of severity (*P* < 0.0001, mild, moderate, and severe CD). The majority of doses given (95%) were 400 U or less; 5% of doses given were >400 U. Injection doses and intervals between injections were at the full discretion of the physician. The median time interval between injections was 94.0–97.5 days for the study cohort and 93.0–98.0 vs. 96.0–97.0 days for patients who were naïve vs. non-naïve to BoNTs, respectively.

### Outcomes

**Figure 4:**
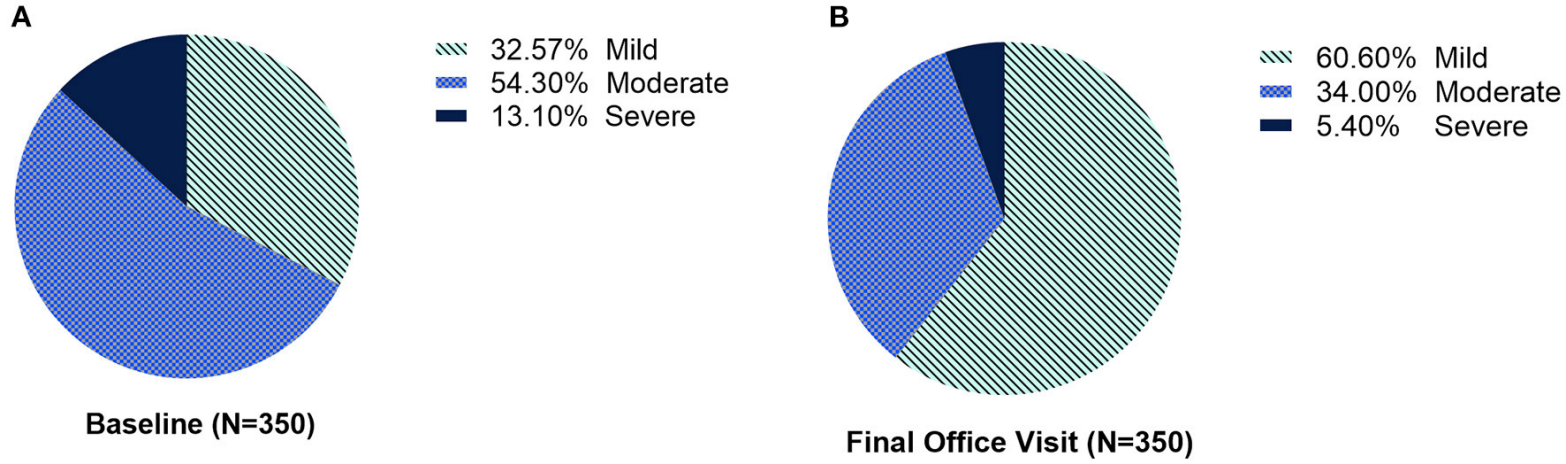
The proportion of patients with CD severity categorized as mild, moderate, or severe, at **(A)** baseline and **(B)** at the final office visit.

Most patients with mild or moderate symptoms (all presentation subtypes combined) maintained or improved their severity scores after each injection. Overall, there was a shift to a lower severity level from baseline to the final office visit (Figures 4A,B). At each treatment, between 40.7 and 65.2% of those categorized as severe shifted to moderate or mild after treatment (Table 2). The improvement in severity overall was similar between patients who were naïve vs. non-naïve to BoNT (Supplementary Figure 1).

**Table 2:** Shifts in CD severity.

		**Injection 2 Severity**		
	**Total**	**Mild**	**Moderate**	**Severe**
	***N** **=*** **350**	***n** **=*** **154**	***n** **=*** **172**	***n** **=*** **24**
**Injection 1 CD Severity**				
Mild	114/350	91/114	21/114	2/114CI>CI
%	32.6 [27.9, 37.6]	79.8 [71.5, 86.2]	18.4 [12.4, 26.5]	1.8 [0.5, 6.2]
Moderate	190/350	58/190	126/190	6/190
% [CI]	54.3 [49.0, 59.4]	30.5 [24.4, 37.4]	66.3 [59.3,72.7]	3.2 [1.5, 6.7]
Severe	46/350	5/46	25/46	16/46
% [CI]	13.1 [10.0, 17.1]	10.9 [4.7, 23.0]	54.3 [40.2, 67.8]	34.8 [22.7, 49.2]
		**Injection 3 Severity**		
	**Total**	**Mild**	**Moderate**	**Severe**
	***N** **=*** **350**	***n** **=*** **168**	***n** **=*** **155**	***n** **=*** **27**
**Injection 2 CD Severity**				
Mild	154/350	127/154	27/154	0/154
% [CI]	44.0 [38.9, 49.2]	82.5 [75.7, 87.7]	17.5 [12.3, 24.3]	0 [0.0, 2.4]
Moderate	172/350	40/172	119/172	13/172
% [CI]	49.1 [43.9, 54.4]	23.3 [17.6, 30.1]	69.2 [61.9, 75.6]	7.6 [4.5, 12.5]
Severe	24/350	1/24	9/24	14/24
% [CI]	6.9 [4.7, 10.0]	4.2 [0.2, 20.2]	37.5 [21.2, 57.3]	58.3 [38.8, 75.5]
		**Peak Effect Office Visit 3 / Exit CD Severity**		
	**Total**	**Mild**	**Moderate**	**Severe**
	***N** **=*** **350**	***n** **=*** **212**	***n** **=*** **119**	***n** **=*** **19**
**Injection 3 CD Severity**				
Mild	168/350	151/168	16/168	1/168
% [CI]	48.0 [42.8, 53.2]	89.9 [84.4, 93.6]	9.5 [5.9, 14.9]	0.6 [0.0, 3.3]
Moderate	155/350	59/155	94/155	2/155
% [CI]	44.3 [39.2, 49.5]	38.1 [30.8, 45.9]	60.6 [52.8, 68.0]	1.3 [0.4, 4.6]
Severe	27/350	2/27	9/27	16/27
% [CI]	7.7 [5.4, 11.0]	7.4 [2.1, 23.4]	33.3 [18.6, 52.2]	59.3 [40.7, 75.5]

*[CI] = 95% Confidence Intervals (Wilson method)*.

**Figure 5:**
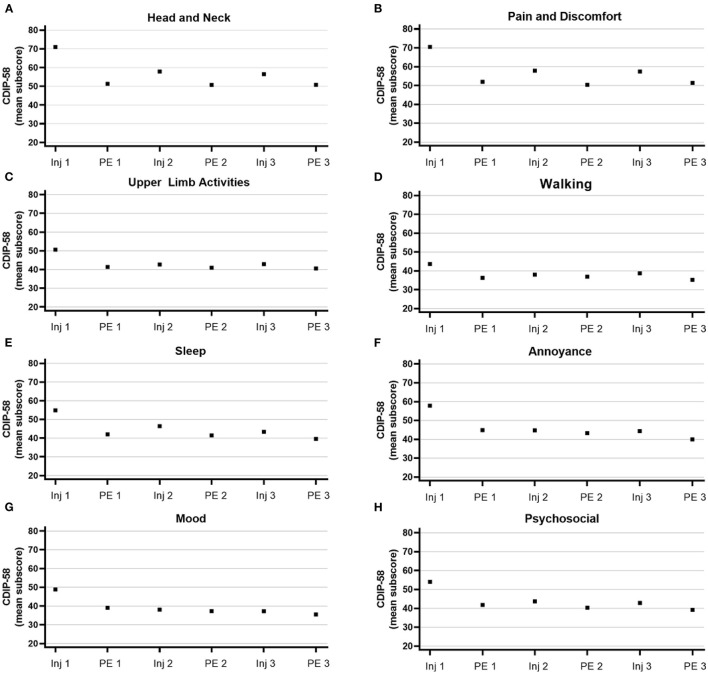
Mean subscores from the Cervical Dystonia Impact Profile-58 (CDIP-58). Subscores for **(A)** head and neck, **(B)** pain and discomfort, **(C)** upper limb activities, **(D)** walking, **(E)** sleep, **(F)** annoyance, **(G)** mood, and **(H)** psychosocial range from 0 to 100. Inj, injection; PE, patient evaluation.

Patients reported sustained improvements across all CDIP-58 subscales (Figure 5). These improvements were relatively similar regardless of prior exposure to BoNTs (Supplementary Figure 2). In patients non-naïve to BoNTs, improvements in CDIP-58 subscales “head and neck” and “pain and discomfort” appeared to wane slightly at later time points.

**Figure 6:**
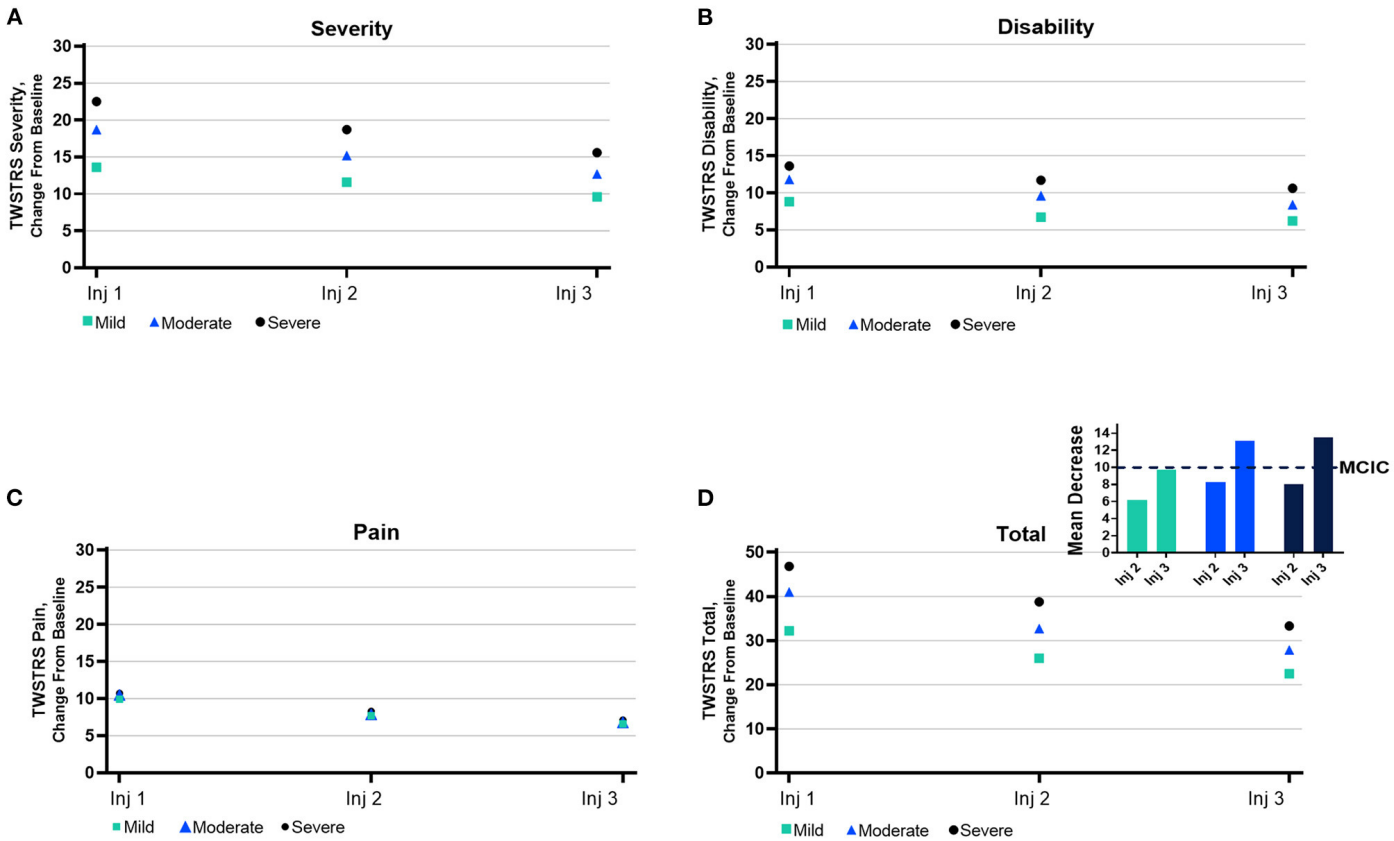
Mean subscores from the Toronto Western Spasmodic Torticollis Rating Scale (TWSTRS). Subscores for TWSTRS domain ranges of **(A)** severity, **(B)** disability, **(C)** pain, and **(D)** total are as follows: severity, 0–35; disability, 0–30; pain, 0–20; and total, 0–85. MCIC, minimal clinically important change.

Physicians reported improvements in mean TWSTRS scores for CD severity, disability, and pain, which were reflected in the improvements in the total mean TWSTRS score (Figure 6). The mean total TWSTRS scores for all presentation subtypes combined demonstrated improvements of 9.7, 13.1, and 13.5 points for those with mild, moderate, and severe CD, respectively (Figure 6). Prior exposure to BoNTs did not diminish improvements in TWSTRS scores, as seen by the relatively similar degree of improvement in mean total TWSTRS scores for naïve (11.8 points) vs. non-naïve (12.4 points) patients (Supplementary Figure 3).

### Safety

No new safety signals were identified. A total of 211 AEs were reported by 112 patients (32.0%) (Table 3). All treatment-related AEs reported by ≥1% of the cohort are shown in Table 3, rank ordered by the percent of patients experiencing events and number of events. In the study cohort, 81 patients (23.1%) reported 139 treatment-related AEs; there were no serious treatment-related AEs. The most common AEs (≥2% of patients) were dysphagia (8.3%), muscular weakness (8.3%), headache (2.9%), and neck pain (2.6%) (Table 3), which were similar between naïve and non-naïve patients (Supplementary Table 2). The most common treatment-related AEs included muscular weakness (8.3%), dysphagia (8.0%), headache (2.3%), and neck pain (2.3%). Four AEs of syncope were reported; 2 were considered serious, and none were treatment related. Two SAEs of hip fracture were reported; neither was treatment related (Table 3).

**Table 3:** AEs, Treatment-Related AEs, and Serious AEs.

	**Patients (*****N** **=*** **350)**	
	**Patients, *n* (%)**	**Events, n**
**AEs**	112 (32)	211
**AEs with incidence** **≥1%**		
*Dysphagia*	29 (8.3)	35
*Muscular weakness*	29 (8.3)	34
*Headache*	10 (2.9)	12
*Neck pain*	9 (2.6)	9
*Injection site pain*	6 (1.7)	6
*Musculoskeletal pain*	5 (1.4)	5
*Musculoskeletal stiffness*	4 (1.1)	4
*Syncope*	4 (1.1)	4
**Treatment-related AEs**	81 (23.1)	139
**Treatment-related AEs with incidence** **≥1%**		
*Muscular weakness*	29 (8.3)	34
*Dysphagia*	28 (8.0)	34
*Headache*	8(2.3)	10
*Neck pain*	8 (2.3)	8
*Injection site pain*	6 (1.7)	6
*Musculoskeletal stiffness*	4 (1.1)	4
**Serious AEs**	9 (2.6)	13
*Hip fracture*	2 (0.6)	2
*Syncope*	2 (0.6)	2
*Bile duct stone*	1 (0.3)	1
*Chest pain*	1 (0.3)	1
*Gastritis*	1 (0.3)	1
*Intestinal mass*	1(0.3)	1
*Loss of consciousness*	1 (0.3)	1
*Orthostatic hypotension*	1 (0.3)	1
*Pneumonia*	1 (0.3)	1
*Polycythemia vera*	1 (0.3)	1
*Skin laceration*	1 (0.3)	1
**Treatment-related serious AEs**	0	0

*AE, adverse event*.

## Discussion

This secondary cohort analysis from CD PROBE demonstrates that 3 repeat treatments with onabotA at intervals consistent with labeling attenuated disease severity, regardless of prior botulinum toxin exposure. After 3 treatment cycles with onabotA, nearly twice as many patients overall were categorized as mild compared to the number categorized as mild at baseline, and less than half as many patients were categorized as severe after 3 treatment cycles compared to the number categorized as severe at baseline. At each treatment visit, patients with the highest severity scores shifted to a lower score after treatment; most patients with mild or moderate severity maintained or demonstrated reductions in disease severity after each treatment. Patients with prior exposure to BoNTs (non-naïve patients) showed incremental improvements across all assessments, indicating that benefits can be achieved even in patients with prior exposure to BoNTs.

Although controlled clinical studies on the treatment of CD with onabotA have been published (40–43), real-world studies such as CD PROBE can provide new insights to inform clinical strategies and optimize patient care. CD PROBE is a multicenter, prospective, clinical trial registry that followed patients with CD who had been treated with dosages and at intervals determined by physicians. This cohort study represents the clinical heterogeneity of patients with CD, including those with presentation subtypes that are typically more difficult to treat, such as anterocollis and retrocollis.

This real-world evidence demonstrates a potential common approach by physicians in the treatment of CD with botulinum toxin. CD presentation subtype (and severity) impacted onabotA utilization. CD subtype frequency differed by CD severity, with torticollis the most common subtype presentation in those with severe CD, followed by laterocollis. For those with torticollis and laterocollis, there was a trend toward increased dosing at later time points. There were far fewer cases of anterocollis, retrocollis, or other types of CD in this cohort study. Overall, those with the most severe disease received higher doses of onabotA than those with milder disease. It is likely that physicians aimed to select the minimal dose that would provide a meaningful change for their patients without side effects (44, 45).

It was observed that non-naïve patients received higher doses than those naïve to BoNT, a difference that is consistent with onabotA labeling (46) and a previous study showing that dosing in patients naïve to BoNT generally increases over the first year and plateaus thereafter (47). In the current study, a relatively greater proportion of non-naïve patients than naïve patients shifted to a lower severity after treatment. Although it is possible that higher doses given to non-naïve patients contributed to a more pronounced shift in severity, there are few published studies, if any, that examine variable dosing by prior exposure to BoNT/A. Differences in severity shifts by prior exposure to BoNTs are not likely related to treatment intervals, as these were relatively similar between the two groups (with intervals consistent with label guidance). The majority (95%) of doses given were consistent with labeling; a small proportion of doses (5%) were above the recommended labeled maximum dose (46). Both dosing levels and judicious targeting of muscles for injection can impact effectiveness of treatment with BoNTs. In this study, the dosing and targeting was at the full discretion of the physician. Successful muscle selection requires clinicians to discriminate between pathologically active muscles and compensatory muscle activity, as well as protective posturing (48). Thus, the variability in dosing likely reflects physicians' selection of the appropriate dose for each patient's specific clinical need and individualized approaches to dosing that provide meaningful clinical improvement while avoiding potential AEs.

To determine the effectiveness of treatment, patient- and physician-reported outcomes were collected using CDIP-58 and TWSTRS, respectively. In addition, physicians assessed CD severity in patients at each visit. The improvements noted by physicians in mean total TWSTRS scores after completion of 3 treatments with onabotA are consistent with the difference of ≥10 points needed to achieve a minimal clinically important change (MCIC) (44, 49, 50); however, individual results varied. Despite noticeable initial improvements in mean TWSTRS scores after the first treatment, mean improvements in TWSTRS did not approach or exceed MCIC levels until after the third treatment for this cohort of patients. The mean improvements noted by patients in CDIP-58 measures (head and neck symptoms, pain and discomfort, upper limb activities, walking, sleep, annoyance, mood, and psychosocial functioning measures) are consistent with improvements noted by physicians in mean TWSTRS scores and physicians' assessments of CD severity. Because only those who completed all treatments and associated visits are included in this cohort study, results must be interpreted with caution.

Up to 90% of patients with CD report that CD-associated neck pain negatively impacts their quality of life (11, 13). Posturing (including constant head turning) is strongly associated with pain, often accompanied by muscle spasms (1). Specific head and neck postures seen in various CD presentation subtypes are associated with differing degrees of pain (51). The improvements in physician- and patient-reported outcomes (TWSTRS and CDIP-58) identified in this study demonstrate that treatment with onabotA at consistent intervals can reduce neck pain associated with CD. These improvements are consistent with a recent study demonstrating that treatment with onabotA resulted in improvements in all CDIP-58 subscales, and in particular, head and neck symptoms (52). Treatments that lessen the pain associated with CD have the potential to positively impact patients' quality of life in many areas, including employment status, as patients with moderate to severe pain due to CD are significantly more likely to cease working due to CD (11).

These real-world data provide further evidence of the efficacy and safety of onabotA for the treatment of CD (35) for a variety of CD presentation subtypes, regardless of prior exposure to BoNTs and potentially pre-existing comorbid dysphagia. Although dysphagia is a commonly reported AE after treatment of CD with BoNTs, pre-existing dysphagia in patients with CD ranges from 11 to 68%, suggesting a predisposition toward dysphagia in those with CD (53–56). According to one study, only half of the patients with CD that had radiographic evidence of abnormal peristalsis prior to treatment with BoNT/A reported symptoms of dysphagia prior to BoNT treatment (56). A robust methodology for assessing dysphagia before and after BoNT treatment may help inform dosing and avoid potential dysphagia-related AEs (57).

Treatment-related dysphagia was 8% in this post-hoc cohort study, which included patients with anterocollis, retrocollis, and those with a history of dysphagia, groups that have been excluded in some recent randomized, controlled trials (ClinicalTrials.gov identifiers: NCT03617367 and NCT03608397). In our overall study population, the rate of dysphagia (6%, *N* = 1041) (35) was similar to that in the current cohort (8%, *N* = 350), both of which were markedly less than label/pivotal trial for onabotA of 19% (43, 46). The relatively lower incidence of dysphagia in the current cohort compared to label may reflect physicians' increased injection competency and ability to tailor treatment for a wide range of patient subtypes as demonstrated in CD PROBE compared to the onabotA pivotal trial. The pivotal trial was performed between 1995 and 1997 (dysphagia, 19%) (43, 46), and CD PROBE was performed between 2009 and 2012 (dysphagia, 8%) (35). The >10 years interim between these trials may have provided physicians the time to gain valuable insights related to injection technique and patient characteristics to optimally treat CD and minimize AEs. When evaluating AEs, it is important to consider the population studied, since excluding those with anterocollis, retrocollis, or a history of dysphagia may result in a relatively lower rate of dysphagia than what would be found without such exclusions, especially in real-world clinic settings. In the current study, onabotA demonstrated an acceptable safety profile, consistent with labeling (46), with no new safety signals identified.

## Limitations

There are several limitations of this study. The open-label design of CD PROBE requires data to be interpreted with caution. The discontinuations from CD PROBE, which were substantial, have been discussed previously (35). In brief, roughly 60% of patients enrolled in CD probe completed all 3 treatments; however, only 48% of those enrolled completed the final office visit and associated assessments (35). Of those initially enrolled, 134 patients who received all 3 treatments did not return for the final office visit; of these, 110 patients were lost to follow up after the third office visit, prior to the final office visit and assessment. Since the final office visit did not involve treatment, patients may not have been motivated to return for the final visit needed to complete final assessments. In addition, some patients might have discontinued treatment if the first treatment was not covered by insurance. It is important to recognize that those who completed the study more than likely had a positive response, resulting in an implicit bias favoring positive outcomes. Those with positive outcomes associated with previous BoNT treatments may also have been predisposed to participate in the CD PROBE study. Due to physicians' autonomy in selecting the dose, frequency, and dilution injection, and the observational nature of this study, trends for this study cohort may not reflect individual dosing trends. Results must be interpreted with caution, given the poor study retention rate and the secondary nature of this analysis.

Nevertheless, it is important to understand who adheres to treatment by severity, subtype, and prior BoNT exposure. The severity shifts seen in this study reflect the population of patients that adhere to treatment. In practice settings, physicians' selection of the dose based on each patient's needs, considering disease severity, prior exposure, and tolerability, resulted in shifts to a lower severity for most patients with moderate to severe disease.

## Conclusion

CD severity impacted presentation subtype frequency and onabotA utilization in this study cohort. Dosing generally increased over time and with increasing disease severity for torticollis, laterocollis, and retrocollis, with relatively higher dosages given to patients who were non-naïve to BoNTs for CD. Of those patients with the highest severity scores, between 40.7 and 65.2% shifted to a lower severity score after each of the 3 injection cycles. At the end of the study, nearly twice as many patients were categorized as mild compared to baseline, and less than half as many patients were categorized as severe compared to baseline. In this real-world study, treatment with onabotA attenuated disease severity and was well tolerated by patients, regardless of prior BoNT exposure.

## Data Availability Statement

AbbVie is committed to responsible data sharing regarding the clinical trials we sponsor. This includes access to anonymized, individual, and trial-level data (analysis data sets), as well as other information (eg, protocols, clinical study reports, or analysis plans), as long as the trials are not part of an ongoing or planned regulatory submission. This includes requests for clinical trial data for unlicensed products and indications. These clinical trial data can be requested by any qualified researchers who engage in rigorous, independent, scientific research, and will be provided following review and approval of a research proposal, Statistical Analysis Plan (SAP), and execution of a Data Sharing Agreement (DSA). Data requests can be submitted at any time after approval in the US and Europe and after acceptance of this manuscript for publication. The data will be accessible for 12 months, with possible extensions considered. For more information on the process or to submit a request, visit the following link: https://www.abbvie.com/our-science/clinical-trials/clinical-trials-data-and-information-sharing/data-and-information-sharing-with-qualified-researchers.html.

## Ethics Statement

The studies involving human participants were reviewed and approved by Institutional Review Boards at each participating institution. The full list is available in the supplementary material (Supplementary Table 3) The patients/participants provided their written informed consent to participate in this study.

## Author Contributions

All authors listed have made a substantial, direct, and intellectual contribution to the work and approved it for publication.

## Funding

AbbVie funded this trial and participated in the trial design, research, analysis, data collection, interpretation of data, and the review and approval of the publication. All authors had access to relevant data and participated in the drafting, review, and approval of this publication. Neither honoraria nor any other form of compensation was provided for authorship. Medical writing was provided by Anita Preininger, Ph.D., of AbbVie; editorial assistance was provided by Angela T. Hadsell of AbbVie.

## Conflict of Interest

PA has served as a speaker/consultant for AbbVie, Acadia, Accorda, Adamas Pharmaceuticals, Amneal, Kyowa Kirin, Sunovion, and US WorldMeds. RB serves as an associate editor for Neurology: Clinical Practice; performs botulinum toxin injections at the University of Rochester (40% effort); serves/has served on scientific advisory boards for Allergan, Ipsen, Merz, and Revance; receives research support from Fox Foundation, NIH (*via* NINDS, ORDR: Dystonia Coalition Projects, Site PI), Revance, and Vaccinex; consultant for Oscine Corporation; consultant for AbbVie and independent rater for AbbVie's ELATE trial; receives fees as section editor and holds stock options in VisualDx; and has served as an expert witness in legal proceedings including malpractice, not involving commercial entities. HM has served as consultant for AbbVie, Acadia, Adamas Pharmaceuticals Inc., Amneal Pharmaceuticals LLC, Lunbeck LLC, Merz, Neurocrine Biosciences, Sunovion, TEVA Pharmaceuticals, UCB, and US World Meds. MSc is the founder of MS Biostatistics, LLC, and was formerly an employee of MedNet Solutions Inc., which was contracted by Allergan to provide biostatistical services for the study. AZ and MSa are employees of AbbVie and may hold AbbVie stock. AP has served as a consultant and speaker for Allergan (an AbbVie company), Ipsen, and as a consultant for Revance. He has received research funding for clinical trials from Allergan, Ipsen, and Revance. Medical writing was provided by A. Preininger, an employee of AbbVie. Editorial assistance was provided by A. Hadsell, an employee of AbbVie. The authors declare that this study received funding from AbbVie. The funder had the following involvement in the study: trial design, research, analysis, data collection, interpretation of data, and the review and approval of the publication.

## Publisher's Note

All claims expressed in this article are solely those of the authors and do not necessarily represent those of their affiliated organizations, or those of the publisher, the editors and the reviewers. Any product that may be evaluated in this article, or claim that may be made by its manufacturer, is not guaranteed or endorsed by the publisher.
