# Supplementary material for: OnabotulinumtoxinA Dosing, Disease Severity, and Treatment Benefit in Patients With Cervical Dystonia: A Cohort Analysis From CD PROBE
Source: Front Neurol. 2022 Jun 30;13:914486. doi: 10.3389/fneur.2022.914486 (PMC9281448; doi:10.3389/fneur.2022.914486)
Supplement: Supplementary file 1 [file Data_Sheet_1.docx]

Supplementary Material

| **Supplemental Table 1. Demographics and Disease Characteristics by Prior Exposure to BoNT (N=350)** | | | | | | | | | |
| --- | --- | --- | --- | --- | --- | --- | --- | --- | --- |
|  |  | **Naïve (n=212)** | | | | **Non-Naïve (n=138)** | | | |
| Age, years, mean (SD) |  | 57.8 | (15.7) | | 56.7 | | (13.0) | |  |
| Female, n (%) |  | 155 | | (73.1) | | 107 | | (77.5) | |
| Race/ethnicity, n (%) |  |  | |  | |  | |  | |
| *White* |  | 201 | | (94.8) | | 130 | | (94.2) | |
| *Hispanic* |  | 5 | | (2.4) | | 3 | | (2.2) | |
| *Asian* |  | 4 | | (1.9) | | 2 | | (1.4) | |
| *Black* |  | 2 | | (0.9) | | 3 | | (2.2) | |
| Time from CD onset to CD diagnosis, |  |  | |  | |  | |  | |
| years, mean (SD) |  | 5.7 | | (8.1) | | 4.0 | | (7.0) | |
| Past treatments, n (%) |  |  | |  | |  | |  | |
| *Muscle resection surgery* |  | 0 | |  | | 0 | |  | |
| *Phenol injection* |  | 0 | |  | | 0 | |  | |
| *Deep brain stimulation* |  | 0 | |  | | 2 | | (1.4) | |
| *Thalamotomy* |  | 0 | |  | | 0 | |  | |
| *Surgical denervation* |  | 1 | | (0.5) | | 5 | | (3.6) | |
| *None of the above* |  | 211 | | (99.5) | | 131 | | (94.9) | |
| CD, cervical dystonia; SD, standard deviation | | | |  | |  | |  | |

| **Supplemental Table 2. Most Common AEs (>2% of patients), Naïve vs Non-Naïve** | | | |
| --- | --- | --- | --- |
| **AE, n (%)** | **Naïve (n=212)** | **Non-Naïve (n=138)** | **Total (N=350)** |
| Muscular weakness |  |  |  |
| *All* | 16 (7.5) | 13 (9.4) | 29 (8.3) |
| *Treatment-related* | 16 (7.5) | 13 (9.4) | 29 (8.3) |
| Dysphagia |  |  |  |
| *All* | 15 (7.1) | 14 (10.1) | 29 (8.3) |
| *Treatment-related* | 14 (6.6) | 14 (10.1) | 28 (8.0) |
| Headache |  |  |  |
| *All* | 7 (3.3) | 3 (2.2) | 10 (2.9) |
| *Treatment-related* | 6 (2.8) | 2 (1.4) | 8 (2.3) |
| Neck pain |  |  |  |
| *All* | 7 (3.3) | 2 (1.4) | 9 (2.6) |
| *Treatment-related* | 6 (2.8) | 2 (1.4) | 8 (2.3) |
| AE, adverse event |  |  |  |

**Supplemental Table 3**

| **Center Name** | **IRB Name** |
| --- | --- |
| Advanced Neurosciences Research, LLC, Fort Collins, CO | MaGil Institutional Review Board (MaGil IRB) |
| Advanced Spine and Rehab, Morristown, TN | MaGil Institutional Review Board (MaGil IRB) |
| Albany Medical College, Albany, NY | Western Institutional Review Board (WIRB) |
| Baylor College of Medicine, Houston, TX | Baylor College of Medicine Institutional Review Board (BCM IRB) |
| Houston VA Medical Center PADRECC, Houston, TX | Baylor College of Medicine Institutional Review Board (BCM IRB) |
| Booth Gardner Parkinson's Care Center, Kirkland, WA | Western Institutional Review Board (WIRB) |
| California Headache & Pain Center, Burbank, CA | MaGil Institutional Review Board (MaGil IRB) |
| Charlotte Neurological Services, Port Charlotte, FL | MaGil Institutional Review Board (MaGil IRB) |
| Child Neurology Center, Gulf Breeze, FL | MaGil Institutional Review Board (MaGil IRB) |
| Clinical Neurosciences of Tampa Bay, Clearwater, FL | MaGil Institutional Review Board (MaGil IRB) |
| Crescent Moon Research, Murrells Inlet, SC | MaGil Institutional Review Board (MaGil IRB) |
| Dent Neurologic Institute, Amherst, NY | MaGil Institutional Review Board (MaGil IRB) |
| Eastern CT Neurology Specialists, Manchester, CT | MaGil Institutional Review Board (MaGil IRB) |
| Florida Center for Headache and Sports Neurology, Port St. Lucie, FL | MaGil Institutional Review Board (MaGil IRB) |
| George Washington Medical Faculty Associates, Washington, DC | Western Institutional Review Board (WIRB) |
| Georgetown University Hospital, Washington, DC | Georgetown University Institutional Review Board |
| Georgia Health Sciences University, Augusta, GA | Medical College of Georgia Institutional Review Board |
| Henry Ford Hospital, Bloomfield, MI | Henry Ford Health System Institutional Review Board |
| Hershey Medical Center, Hershey, PA | Penn State Hershey Institutional Review Board |
| Hospital for Joint Diseases, New York, NY | NYU School of Medicine Institutional Review Board |
| Indiana University School of Medicine, Carmel, IN | Indiana University - Purdue University Indianapolis IRB-02 |
| Intercoastal Medical Group, Sarasota, FL | MaGil Institutional Review Board (MaGil IRB) |
| International Clinical Research Institute (ICRI) Inc., Overland Park, KS | MaGil Institutional Review Board (MaGil IRB) |
| Island Neurological Associates, PA, Plainville, NY | MaGil Institutional Review Board (MaGil IRB) |
| John Hopkins University, Baltimore, MD | John Hopkins Medicine Institutional Review Board (JHM IRB) |
| Kansas City Bone & Joint Clinic, Overland Park, KS | MaGil Institutional Review Board (MaGil IRB) |
| Lahey Clinic, Burlington, MA | Lahey Clinic Institutional Review Board |
| Lehigh Valley Hospital - Neuroscience Pain Research, Allentown, PA | Lehigh Valley Hospital Institutional Review Board |
| Loma Linda University. Loma Linda, CA | Loma Linda University Institutional Review Board |
| Mankato Clinic, Mankato, MN | MaGil Institutional Review Board (MaGil IRB) |
| Mayo Clinic Arizona, Scottsdale, AZ | Mayo Clinic Institutional Review Board |
| Medical College of Georgia, Augusta, GA | Medical College of Georgia Institutional Review Board |
| Medical University of SC, Charleston, SC | Medical University of South Carolina Instituional Review Board |
| Methodist Neurological Institute, Houston, TX | The Methodist Hospital Research Institute IRB 2 |
| Michigan Neurology Institute, Detroit, MI | MaGil Institutional Review Board (MaGil IRB) |
| Michigan State University, East Lansing, MI | Michigan State University Institutional Review Board |
| MIMA Neurology, Melbourne, FL | MaGil Institutional Review Board (MaGil IRB) |
| Mount Sinai School of Medicine, New York, NY | BRANY Institutional Review Board |
| Negroski, Sutherland and Hanes Neurology, LLP, Sarasota, FL | MaGil Institutional Review Board (MaGil IRB) |
| Neurology & Neurodiagnostics of Alabama, Alabaster, AL | MaGil Institutional Review Board (MaGil IRB) |
| Neurology Specialist located in Yorktown Heights, NY | MaGil Institutional Review Board (MaGil IRB) |
| Neurology Specialists of Dallas, Dallas, TX | MaGil Institutional Review Board (MaGil IRB) |
| North American Partners in Pain Management, Valley Stream, NY | MaGil Institutional Review Board (MaGil IRB) |
| North Central Texas PM&R, Frisco, TX | MaGil Institutional Review Board (MaGil IRB) |
| Northshore Clinical Associates, Erie, PA | MaGil Institutional Review Board (MaGil IRB) |
| Northwest Neurological, PLLC, Spokane, WA | MaGil Institutional Review Board (MaGil IRB) |
| Northwestern University, Chicago, IL | Northwestern University Institutional Review Board |
| Ortho Neuro/New Albany Surgical Hospital Foundation, New Albany, OH | MaGil Institutional Review Board (MaGil IRB) |
| Parkinson's Disease and Movement Disorders Center of Boca Raton, Boca Raton, FL | MaGil Institutional Review Board (MaGil IRB) |
| Peninsula Health Center, San Mateo, CA | MaGil Institutional Review Board (MaGil IRB) |
| Puget Sound Neurology Movement Disorder Clinic, Tacoma, WA | MaGil Institutional Review Board (MaGil IRB) |
| Regional Rehabilitation Center, PLLC, McMinnville, TN | MaGil Institutional Review Board (MaGil IRB) |
| Rochester General Hospital, Rochester, NY | Rochester General Hospital |
| Rocky Mountain Movement Disorders Center, Englewood, CO | MaGil Institutional Review Board (MaGil IRB) |
| Rush University Medical Center, Chicago, IL | Rush University Medical Center Institutional Review Board |
| Seacoast Physiatry, Portsmouth, NH | MaGil Institutional Review Board (MaGil IRB) |
| Siskin Hospital for Physical Rehabilitation, Chattanooga, TN | MaGil Institutional Review Board (MaGil IRB) |
| Springfield Neurology Associates, Springfield, MA | MaGil Institutional Review Board (MaGil IRB) |
| St. Luke's Neurological Associates, Allentown, PA | St. Luke's Hospital & Health Network Institutional Review Board |
| Sun Health Research Institute, Sun City, AZ | Western Institutional Review Board (WIRB) |
| Sunrise Medical Research, Inc., Lauderdale Lakes, FL | MaGil Institutional Review Board (MaGil IRB) |
| The Innovative Clinical Research Center, Alexandria, VA | MaGil Institutional Review Board (MaGil IRB) |
| The Parkinson's and Movement Disorder Institute, Fountain Valley, CA | MaGil Institutional Review Board (MaGil IRB) |
| The Parkinson's and Movement Disorders Clinic, Southfield, MI | MaGil Institutional Review Board (MaGil IRB) |
| Thomas Jefferson University, Philadelphia, PA | Thomas Jefferson University Institutional Review Board |
| University of Alabama at Birmingham, Birmingham, AL | The University of Alabama at Birmingham Institutional Review Board |
| University of Arizona, Tucson, AZ | University of Arizona Institutional Review Board (IRB 2) |
| University of California Davis Medical Center, Sacramento, CA | University of California Davis Institutional Review Board |
| University of Florida Movement Disorders Center, Gainesville, FL | Western Institutional Review Board (WIRB) |
| University of Florida - Jacksonville, Jacksonville, FL | Western Institutional Review Board (WIRB) |
| University of Kansas Medical Center, Kansas City, KS | The University of Kansas Medical Center Institutional Review Board |
| University of Miami, Miami, FL | University of Miami Institutional Review Board |
| University of Rochester Neurology, Rochester, NY | University of Rochester Research Subject Review Board |
| University of South Florida, Tampa, FL | University of South Florida Institutional Review Board |
| University of Texas Health Science Center - San Antonio, San Antonio, TX | University of Texas Health Science Center San Antonia Institutional Review Board |
| University of Vermont, Burlington, VT | University of Vermont Institutional Review Board |
| UT Southwestern Medical Center, Dallas, TX | UT Southwestern Medical Center Institutional Review Board |
| VUMC Dept. of Neurology - Movement Disorders Division, Nashville, TN | Vanderbilt University Institutional Review Board |
| Wake Forest University Health Sciences, Winston-Salem, NC | Wake Forest Institutional Review Board |
| Wellspan Neurology, York, PA | Wellspan Health Institutional Review Board |
| West Virginia University, Morgantown, WV | West Virginia University Institutional Review Board |
| Yale University School of Medicine, New Haven, CT | Yale University Human Investigation Committee (HIC) |

**
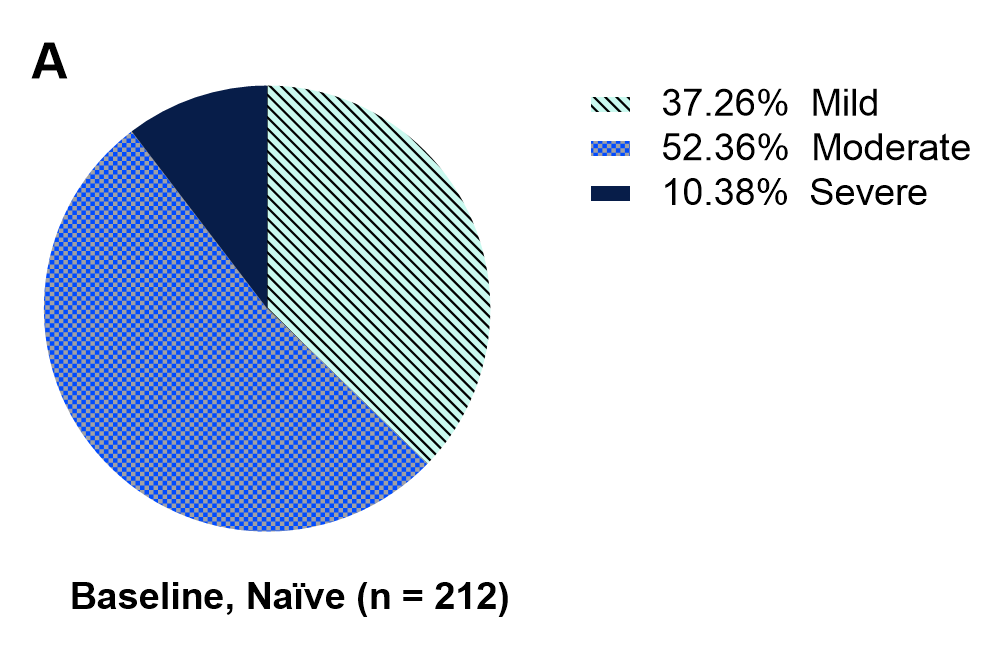
Supplemental Figure 1**

**
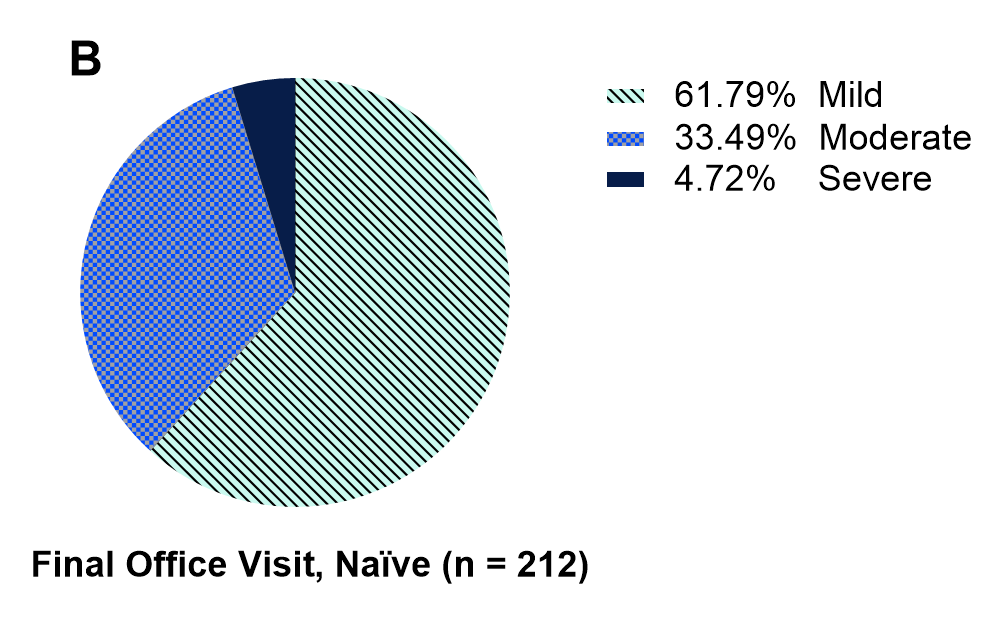
**

**
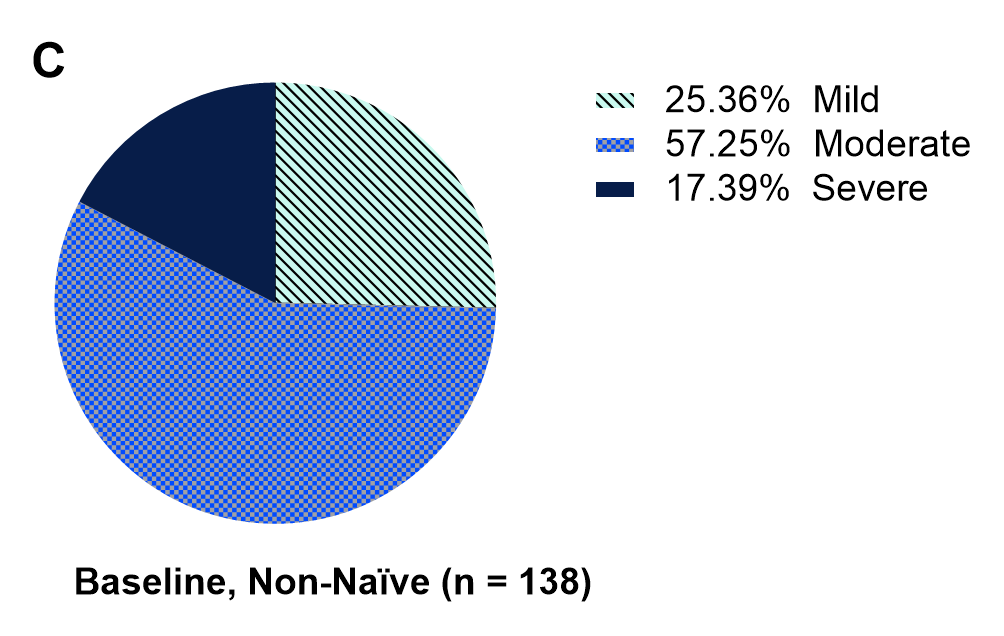

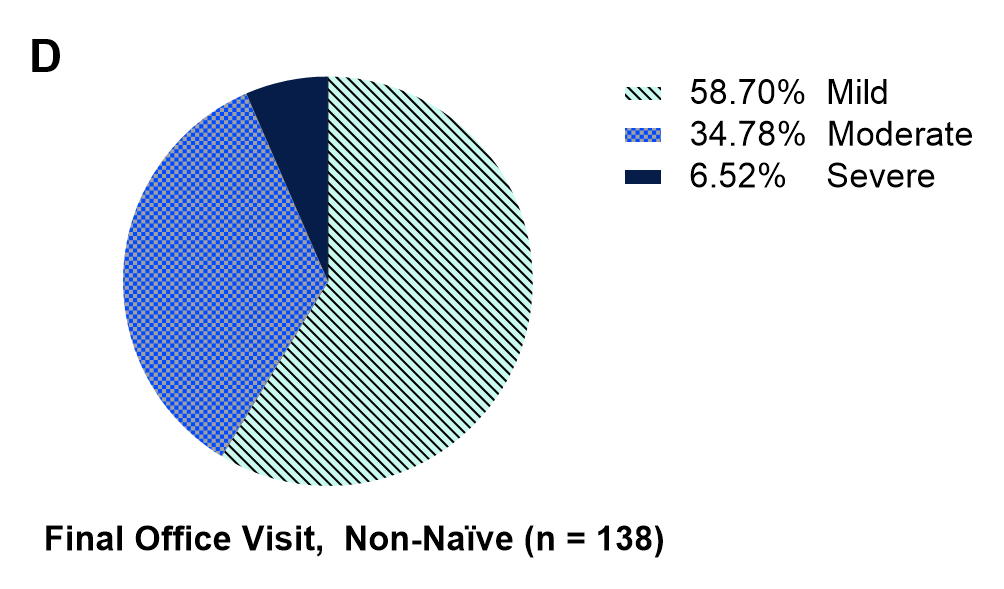
**

**Supplementary Figure 1.** The proportion of patients with CD severity categorized as mild, moderate, or severe, at baseline (A, C) and the final office visit (B, D) for those naïve (A, B) and non-naïve (C, D) to BoNT for CD. Those non-naïve to BoNT received BoNT for CD more than 16 weeks prior to enrollment in the current study.

**
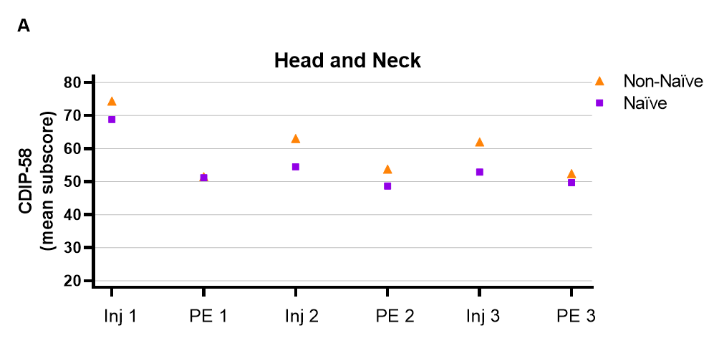
Supplemental Figure 2**


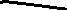


**
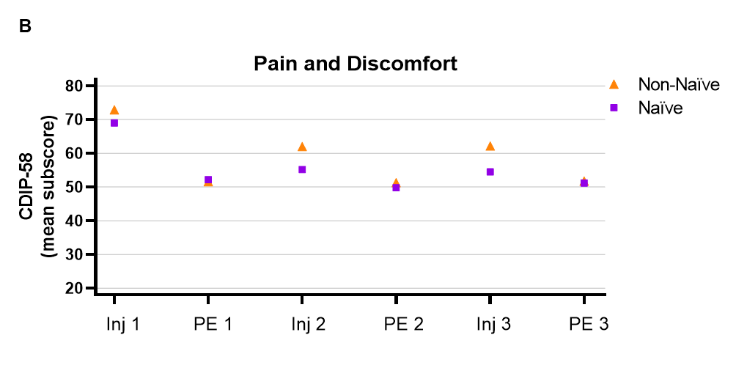
**

**
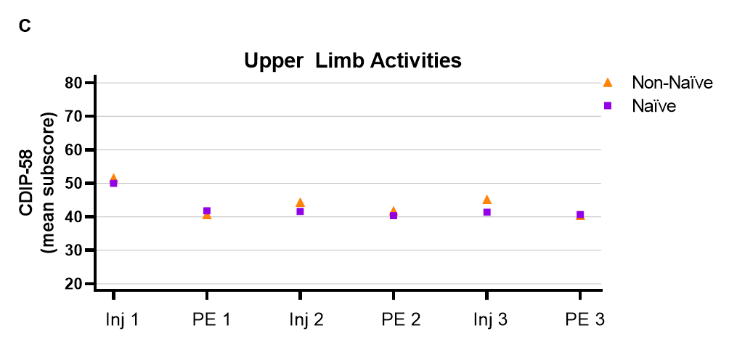

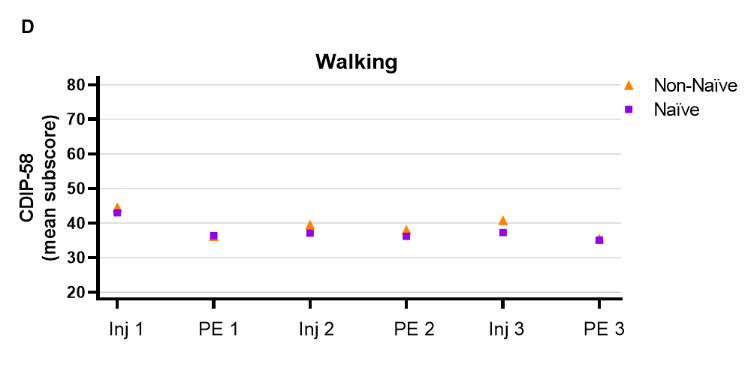
**

**
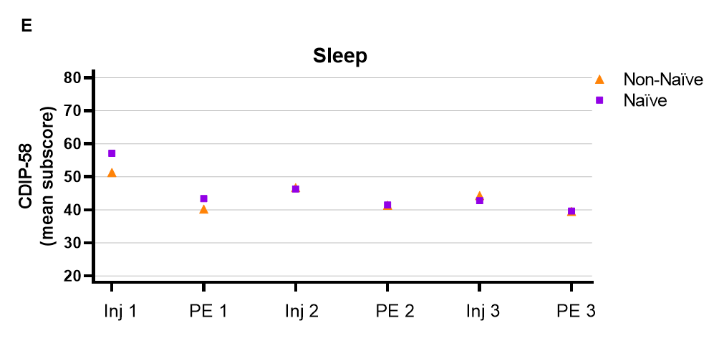

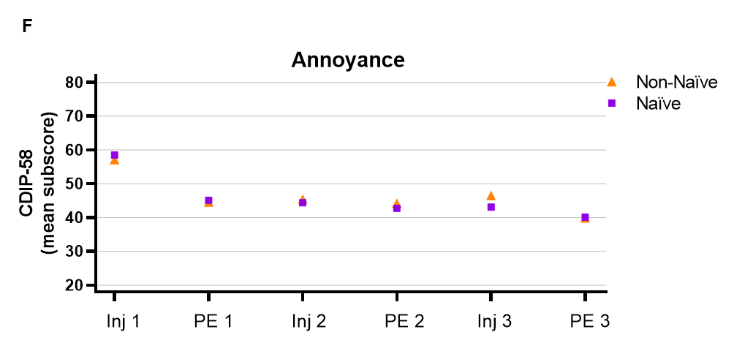
**

**
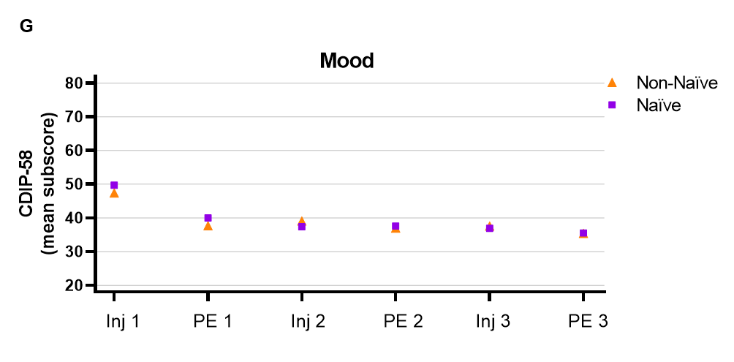

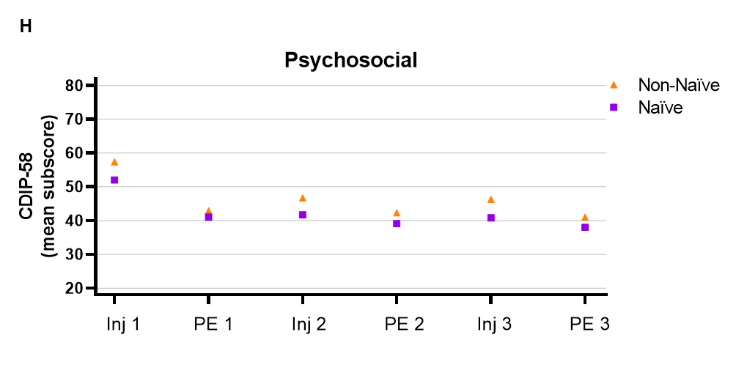
**

**Supplementary Figure 2.** Mean subscores from the Cervical Dystonia Impact Profile-58 (CDIP-58). Subscores for those patients who were naïve (purple) or non-naïve (orange) to BoNT for CD: (A) head and neck, (B) pain and discomfort, (C) upper limb activities, (D) walking, (E) sleep, (F) annoyance, (G) mood, and (H) psychosocial. Scores range from 0 to 100. Inj, injection; PE, patient evaluation.

**Supplemental Figure 3**

**
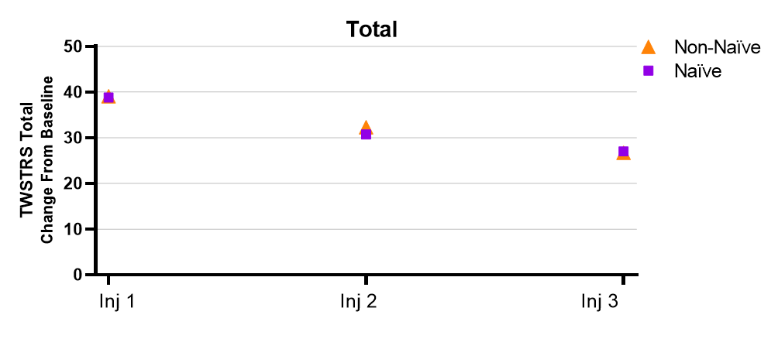
**

**Supplementary Figure 3.** Total score from the Toronto Western Spasmodic Torticollis Rating Scale (TWSTRS, range 0-85) for patients who were naïve (purple) or non-naïve (orange) to BoNT for CD.
